# Study of Morphological Changes in MgH_2_ Destabilized LiBH_4_ Systems Using Computed X-ray Microtomography

**DOI:** 10.3390/ma5101740

**Published:** 2012-09-26

**Authors:** Tabbetha Dobbins, Shathabish NaraseGowda, Leslie G. Butler

**Affiliations:** 1 Department of Physics and Astronomy, Science Hall, Rowan University, Glassboro, NJ 08028, USA; 2 Department of Chemical Engineering, Institute for Micromanufacturing, Louisiana Tech University, Ruston, LA 71270, USA; E-Mail: sna016@latech.edu; 3 Department of Chemistry, 329 Choppin Hall, Louisiana State University, Baton Rouge, LA 71245, USA; E-Mail: lbutler@lsu.edu

**Keywords:** microtomography, MgH_2_, LiBH_4_, destabilized hydrides

## Abstract

The objective of this study was to apply three-dimensional x-ray microtomographic imaging to understanding morphologies in the diphasic destabilized hydride system: MgH_2_ and LiBH_4_. Each of the single phase hydrides as well as two-phase mixtures at LiBH_4_:MgH_2_ ratios of 1:3, 1:1, and 2:1 were prepared by high energy ball milling for 5 minutes (with and without 4 mol % TiCl_3_ catalyst additions). Samples were imaged using computed microtomography in order to (i) establish measurement conditions leading to maximum absorption contrast between the two phases and (ii) determine interfacial volume. The optimal energy for measurement was determined to be 15 keV (having 18% transmission for the MgH_2_ phase and above 90% transmission for the LiBH_4_ phase). This work also focused on the determination of interfacial volume. Results showed that interfacial volume for each of the single phase systems, LiBH_4_ and MgH_2_, did not change much with catalysis using 4 mol % TiCl_3_. However, for the mixed composite system, interphase boundary volume was always higher in the catalyzed system; increasing from 15% to 33% in the 1:3 system, from 11% to 20% in the 1:1 system, and 2% to 14% in the 2:1 system. The parameters studied are expected to govern mass transport (*i.e.*, diffusion) and ultimately lead to microstructure-based improvements on H_2_ desorption and uptake rates.

## 1. Introduction

Many light metal hydrides have been considered for hydrogen storage applications. Among these, the light metal hydride which has demonstrated a higher hydrogen gravimetric capacity at 9 wt % hydrogen is LiBH_4_. This hydride requires 400 °C for hydrogen desorption and has recently been “destabilized” using MgH_2_, which produces the more thermodynamically stable product phase MgB_2_ in the reaction to release 12 wt % H_2_.

(1) $$\text{LiB}{\text{H}}_{4}+\frac{1}{2}\text{Mg}{\text{H}}_{2}↔\text{LiH}+\frac{1}{2}\text{Mg}{\text{B}}_{2}+2{\text{H}}_{2}$$

This destabilization reaction was first demonstrated by Vajo *et al*. in 2005—who reduced the enthalpy of the dehydrogenation reaction by 25 kJ/mol H_2_ over the pure LiBH_4_ [1,2]. By extrapolation, this data implies a lowering in the reaction temperature at 1 bar of H_2_ pressure from 400 °C in pure LiBH_4_ to 225 °C in the destabilized system [1,2]. Reaching the predicted temperatures would require a thorough understanding of interphase reaction rates and interphase boundary volumes. The early concept of destabilized metal hydrides is attributed to Reilly and Wiswall in a study of MgH_2_ with Cu as a destabilizer [3]. Further thermodynamic predictions show that other M(BH_4_)_x_ compounds (where M = Ca, Mg, Al, Li, and Na) should undergo similar destabilization reactions with other borohydrides, amides, and magnesium or silicon hydrides [4,5]. Still, slow dehydrogenation kinetics and reversibility of the destabilized systems remain an issue. Examining phase distributions of two phases, *i.e*., LiBH_4_ and MgH_2_, using 3D imaging will provide the opportunity to understand diffusion and desorption kinetic limitations in the destabilized hydride systems. Likewise, the addition of catalysts, which have been shown to enhance diffusion in the single phase regions [6], is expected to improve overall kinetics and address reversibility issues.

The present study attempts to address the kinetics of destabilized light metal hydride systems by examining hydride microstructures using 3 dimensional imaging (x-ray tomography) in order to determine reactant phase domain size and interphase boundary volume. These parameters will play a critical role for understanding and modeling ionic transport. As well, quantity and distribution of interphase boundaries—which are sites for the reaction of MgH_2_/LiBH_4_ to form LiH/MgB_2_ can be directly measured using 3D imaging [7]. Technological challenges in microtomographic imaging of hydrides to be overcome include: (i) understanding the influence of x-ray energy (in keV) on phase contrast and (ii) utilizing image processing in order to quantify content of phases and interphase boundary volume. Here, we present very preliminary studies which show the possibility for tomographic to overcome such challenges and address microstructure-based issues related to thermodynamic and kinetic limitations within the destabilized hydrides.

## 2. Results and Discussion

### 2.1. 3D Imaging Using Absorption Contrast

**Figure 1:**
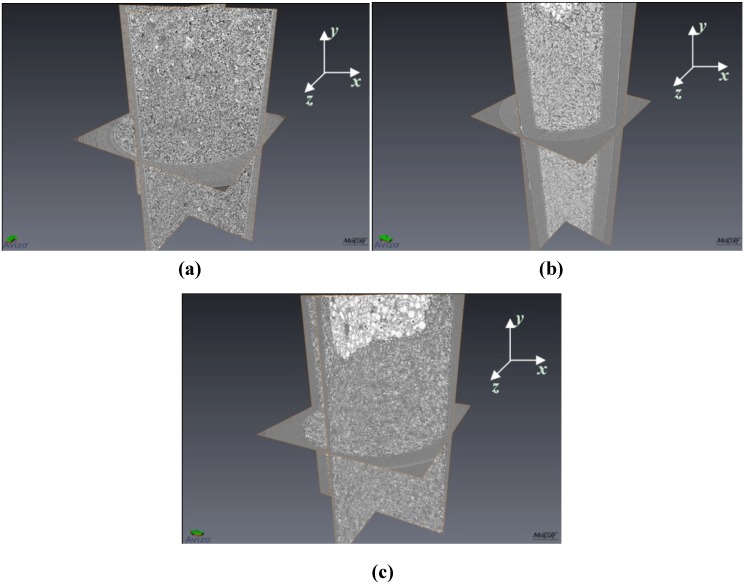
Three dimensional images of (**a**) 4 mol % TiCl_3_ catalyzed LiBH_4_; (**b**) 4 mol % TiCl_3_ catalyzed MgH_2_, and (**c**) 4 mol % TiCl_3_ catalyzed LiBH_4_:MgH_2_ in 1:1 molar ratio. Here only the x-y, y-z, and x-z planes are rendered in the images. For reference, the inner diameter of the tube (seen in the x-z plane) is 1.87 mm. In (**b**) and (**c**) the bright/white areas are tomographic images of the epoxy putty used to seal the samples within the holders (and are not a part of the sample itself). In subsequent sections, image analysis data performed on all individual x-z planar slices is reported.

**Figure 2:**
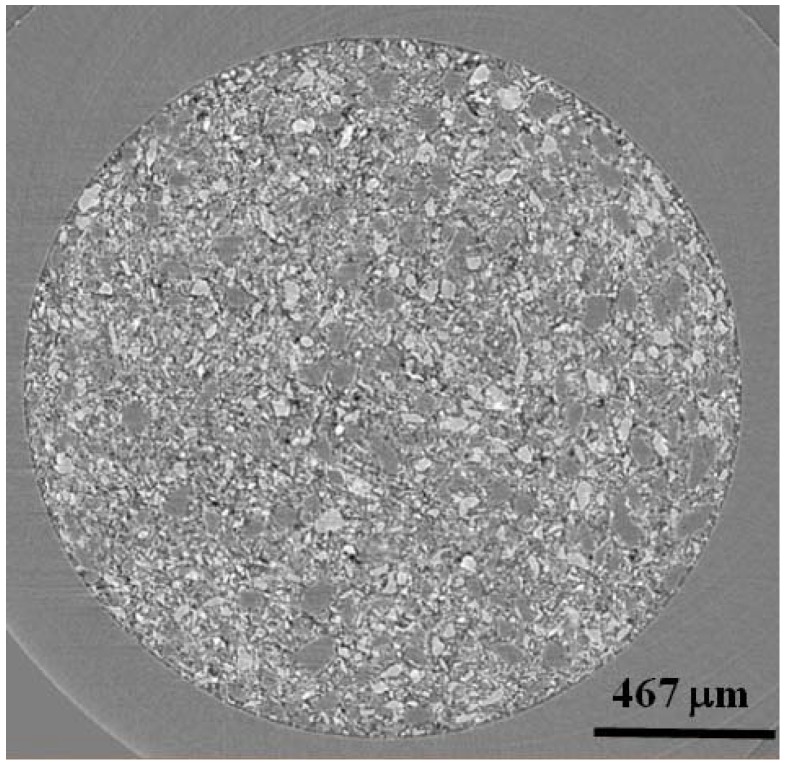
A single x-z oriented slice from 4 mol % TiCl_3_ catalyzed LiBH_4_:MgH_2_ in 1:1 molar ratio. Light contrasting particles are LiBH_4_ and dark contrasting particles are MgH_2_. The black areas are air or void space.

Figure 1 shows equiaxed morphologies for powders of (a) LiBH_4_, (b) MgH_2_, and (c) a 1:1 mixture of LiBH_4_:MgH_2_. Similar morphologies were found for the 1:3 and 2:1 mixtures. Figure 2 shows a single slice (in x-z plane) from the 1:1 mixture of LiBH_4_:MgH_2_. Here, it is clear to see that the phase contrast between LiBH_4_ (above 90% transmission at 15 keV) and MgH_2_ (18% transmission at 15 keV) gives rise to LiBH_4_ particles which appear lighter than the darker MgH_2_ ones. The LiBH_4_ particles are ~50 μm–100 μm in size and the MgH_2_ particles are slightly larger at ~100 μm–150 μm. Because of high transmission for LiBH_4_, sample to detector distances were adjusted to increase edge contrast of the LiBH_4_ phase.

### 2.2. Using Microtomography to Determine Relative Amounts of LiBH_4_ and MgH_2_

**Figure 3:**
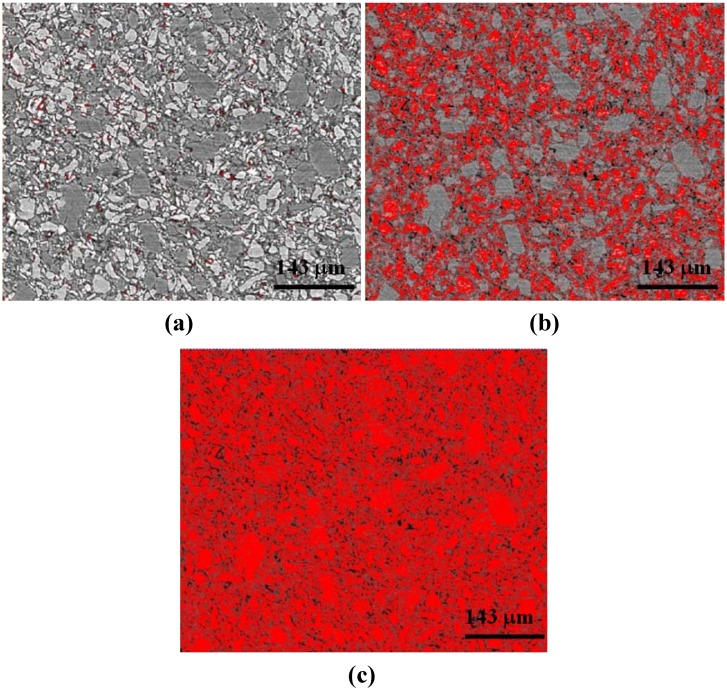
Two dimensional x-z plane slices were used for thresholding. Here the 1:3 LiBH_4_:MgH_2_ (uncatalyzed) sample with (**a**) no thresholding; (**b**) red highlighted pixels corresponding to thresholding for LiBH_4_; and (**c**) red highlighted pixels corresponding to thresholding for both LiBH_4_ and MgH_2_. Taking the difference in volume computed from (**c**) and (**b**), we may determine the volume of MgH_2_. The black areas are air or void space.

Figure 3 shows an example of image analysis thresholds used. Here the 1:3 LiBH_4_:MgH_2_ (uncatalyzed) sample is shown. The thresholded images were derived from histograms of the grey-scale images wherein a narrow peak (corresponding to the lightest grey) denotes the LiBH_4_ phase—whilst a second, broader peak-like feature in the histogram corresponds to the MgH_2_ phase. These two peak-like features showed a slight overlap. For LiBH_4_ thresholding, the levels were selected to capture most of the “narrow” peak (omitting overlap regions). For both LiBH_4_ and MgH_2_ thresholding, the levels were selected to capture both the “narrow” peak and the broadened peak-like features—using the difference in the volumes of those levels computed by Avizio^TM^ software as the MgH_2_ volume. In all cases, images were inspected after the first threshold selection (of the narrow peak) to ensure all highlighted (red) pixels are comprised of LiBH_4_. Images were also inspected upon the second threshold selection (corresponding to both the narrow and broader peaks) to ensure that highlighted pixels correspond to material (not void space). Figure 3a shows images without thresholding and Figures 3b,c show thresholding for the LiBH_4_ phase (selection of the narrow peak) and the combined MgH_2_/LiBH_4_ phases (selection of both peaks), respectively.

After thresholding the images using 2D x-z plane slices, volume fraction data is calculated by Avizo^TM^ software using voltex volume data. Table 1 shows volume data (in μm^3^) computed using pixel count of microtomographic data. Table 2 shows molar ratios calculated from volume fraction data. Results show that experimental determination of relative amount of each phase measured by tomographic imaging did not correctly predict the relative amounts of LiBH_4_ and MgH_2_ used for sample preparation. It is hypothesized that the poor correlations to target compositions prepared at LiBH_4_:MgH_2_ molar ratios of 1:1, 1:3, and 2:1 may have occurred because the high energy ball milling process resulted in particles sizes which are either below the 2 μm spatial resolution of the tomographic imaging or smaller particles settled to the bottom of the sample vial where they were not included in the measurement.

The molar ratio data shown in Table 2 indicates that the majority constituent in each two phase mixture is milled to finer sizes than the smaller constituent. High energy ball milling results in reduced particle sizes with increased mill time. The smallest particles in the system may not have been included in the image sets because of gravitational settling or because of milling to sizes below the spatial resolution of the tomography. Some factors which influence the final particle size are material hardness, initial particle size, and ratio of powder-to-milling media (*i.e*., stainless steel mill balls). In these experiments, LiBH_4_ and MgH_2_ phases were milled simultaneously keeping the ratio of powder-to milling media fixed. It is important to note that when molar ratio of the constituent powders changes—the ratio of powder-to-milling media for each phase is also changed. This variable would determine which phase attrition mills at a higher rate and hence reaches to sizes below the spatial resolution of the tomographic instrument or settles to bottom of sample vial. For example, in the 1:3 LiBH_4_:MgH_2_ (molar ratio) mixed powder system, the stoichiometric starting composition is 0.3 mol LiBH_4_ to 1 mol of MgH_2_—however, the tomographic imaging data erroneously measures 0.46 mol of LiBH_4_ to 1 mol of MgH_2_ (*i.e.*, the imaging data reports too little MgH_2_). Since MgH_2_ is the most abundant phase in the starting powder—its ratio of powder-to-milling media is higher resulting in a higher probability of contact with milling media and a higher rate of particle attrition leading to size reduction. Indeed, this explanation would account for the poor correlations in the 2:1 LiBH_4_:MgH_2_ (molar ratio) mixed powders whereby a higher ratio of powder-to-milling media for LiBH_4_ yields more rapid reduction in particle size for this phase. As a result, imaging data erroneously measures lower than expected amounts of LiBH_4_ (measured at 0.45 mol of LiBH_4_ per 1 mol of MgH_2_). In the case of the 1:1 sample, the measurements show 0.39 mol of LiBH_4_ to 1 mol of MgH_2_ for the uncatalyzed and 0.59 mol of LiBH_4_ to 1 mol of MgH_2_ for the catalyzed system. In both cases (*i.e*., catalyzed and uncatalyzed), the amount of LiBH_4_ was underestimated by these measurements indicating that LiBH_4_ was milled more effectively to smaller sizes. The density of LiBH_4_ is approximately half that of MgH_2_ and so it can be reasoned that, at equal molar amounts, the volume of LiBH_4_ is twice that of the MgH_2_ (leading to more opportunity for the LiBH_4_ phase to encounter the milling media and hence more particle size reduction). Table 2 summarizes this data and includes—in parenthesis—notation of the phase which is most abundant and below spatial resolution. Again, the abundant phase in each mixture is underestimated by the imaging data because of gravitation sedimentation which may have caused it to fall to the bottom of the sample vial where it was excluded from measurement or because during attrition milling it achieved sizes smaller than the spatial resolution of the tomographic instrument.

**Figure 4:**
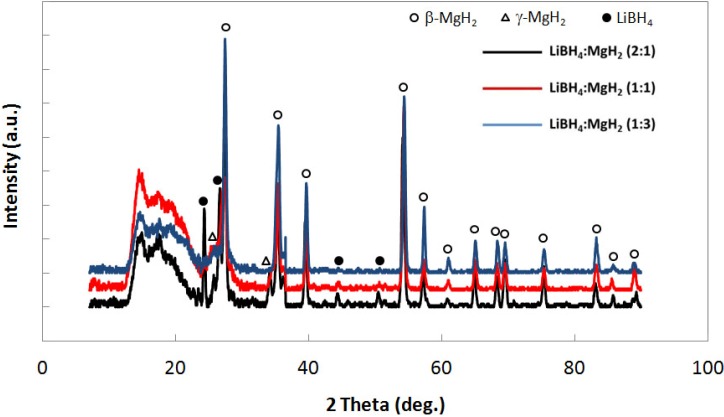
X-ray diffraction for uncatalyzed LiBH_4_:MgH_2_ in 2:1, 1:1 and 1:3 molar ratios.

In all cases of measured compositions, the percent difference between measured and target composition is very large. Therefore, to bring into focus the actual compositions of the three samples, x-ray diffraction measurements were made. Figure 4 shows x-ray diffraction of the uncatalyzed samples and clearly indicates increasing amounts of LiBH_4_ in going from the 1:3, 1:1 and 2:1 (LiBH_4_:MgH_2_) as indicated by the increasing in peak intensity at 2θ~22° and at 2θ~49.98°. By x-ray diffraction, it is confirmed that no desorption product, *i.e*., LiH, is present. LiH diffraction peaks occur at 2θ~38° and 44° [8].

**Table 1:** Total volume of void space, LiBH_4_, and MgH_2_ (in μm^3^) computed from pixel count in computed microtomographic images. These data were used (along with density and molecular weight) to compute molar ratios or molar fractions (reported in Table 2). In computing molar ratios, the void space volume was attributed equally to LiBH_4_ and MgH_2_.

Volume Air	Volume LiBH_4_	Volume MgH_2_
1:3 Catalyzed LiBH_4_:MgH_2_		
1.60 × 10^8^	3.23 × 10^8^	2.26 × 10^8^
1:3 Uncatalyzed LiBH_4_:MgH_2_		
1.71 × 10^8^	1.76 × 10^8^	2.27 × 10^8^
1:1 Catalyzed LiBH_4_:MgH_2_		
1.77 × 10^8^	2.78 × 10^8^	2.57 × 10^8^
1:1 Uncatalyzed LiBH_4_:MgH_2_		
1.04 × 10^8^	7.64 × 10^7^	1.16 × 10^8^
2:1 Catalyzed LiBH_4_:MgH_2_		
1.45 × 10^8^	1.68 × 10^8^	2.49 × 10^8^
2:1 Uncatalyzed LiBH_4_:MgH_2_		
1.46 × 10^8^	1.30 × 10^7^	3.24 × 10^7^

**Table 2:** LiBH_4_:MgH_2_ molar ratios computed from microtomographic image analysis for catalyzed and uncatalyzed systems. Poor correlations between the target molar ratios of 0.3, 1.0 and 2.0 may be explained if high energy ball milling preferentially reduces particle sizes for the most abundant phase in the mixture.

Target LiBH_4_:MgH_2_ Composition	LiBH_4_:MgH_2_ Molar Fractions for Uncatalyzed Samples	LiBH_4_:MgH_2_ Molar Fractions for 4 mol % TiCl_3_ Catalyzed Samples
1:3	0.46	0.73
(0.33 LiBH_4_ molar fraction)	(more MgH_2_ below spatial resolution)	(more MgH_2_ below spatial resolution)
1:1	0.39	0.59
(1.0 LiBH_4_ molar fraction)	(more LiBH_4_ below spatial resolution)	(more LiBH_4_ below spatial resolution)
2:1	0.45	0.41
(2.0 LiBH_4_ molar fraction)	(more LiBH_4_ below spatial resolution)	(more LiBH_4_ below spatial resolution)

### 2.3. Interfacial Volume for Single Phase MgH_2_ and LiBH_4_ and Interphase Volume for 3:1, 1:1, and 2:1 Mixtures of LiBH_4_:MgH_2_

**Figure 5:**
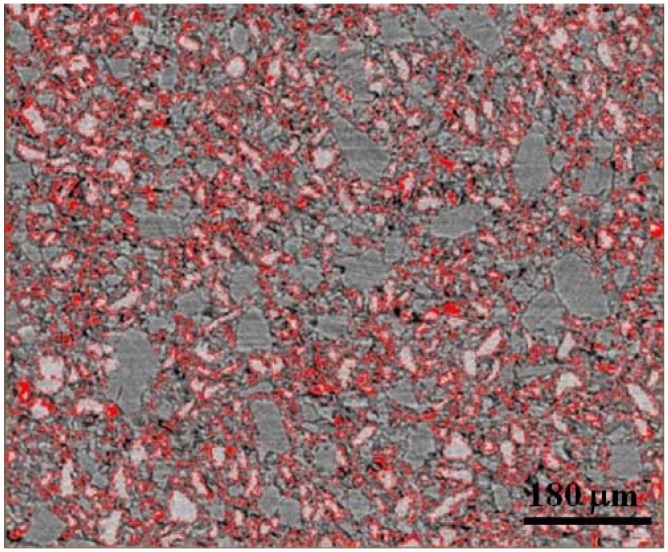
Two dimensional x-z slice used for thresholding edges of the LiBH_4_ phase in the 1:3 LiBH_4_:MgH_2_ (uncatalyzed) sample. Similar threshold levels were selected for computing interfacial boundary volume between LiBH_4_ and MgH_2_ for all samples data.

**Figure 6:**
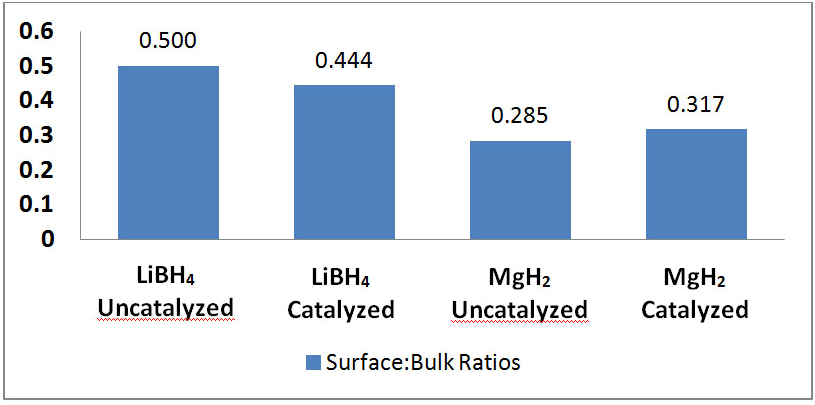
Comparison of interfacial boundary volume for LiBH_4_ (uncatalyzed), LiBH_4_ (catalyzed), MgH_2_ (uncatalyzed), and MgH_2_ (catalyzed). The LiBH_4_ phase contains smaller particles (*i.e.* higher interface volume to bulk volume ratios) than the MgH_2_ phase. No significant trend in interfacial volume for catalyzed and uncatalyzed samples is noted in either the case of pure LiBH_4_ or MgH_2_.

**Figure 7:**
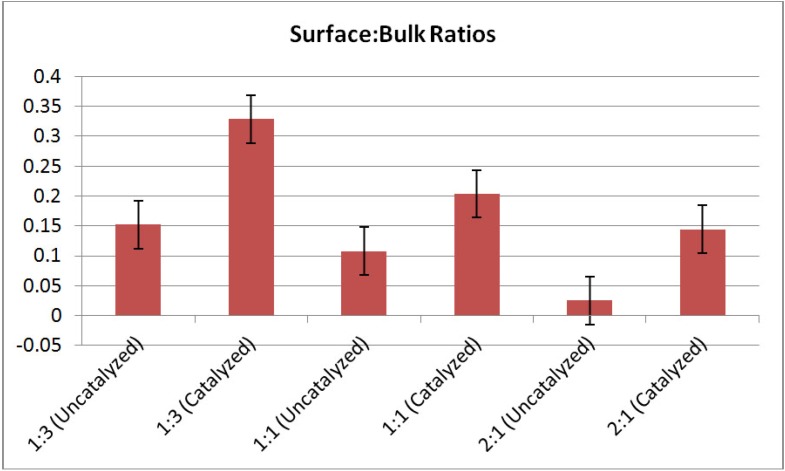
Interphase boundary to bulk volume ratios for LiBH_4_:MgH_2_ mixtures in 1:3, 1:1, and 2:1 molar ratios. Error bars shown are +/−0.04. In all cases, the interphase volume is increased after 4 mol % TiCl_3_ catalyst additions. Again, the interphase volume is computed using the edge thresholding for the LiBH_4_ phase—and so naming these edges as “interphase volume” makes the assumption that each LiBH_4_ particle is surrounded by MgH_2_ (not air or other LiBH_4_ particles).

The tomographic images showed a bright fringe around the edges of the LiBH_4_ particles—and so Avizo^TM^ software could be used to threshold those edges in the mixed powder systems. This may have been possible because of phase contrast yielding brighter edges around LiBH_4_ and optimal edge enhancement for that phase. Within the grey-scale histogram, a very low intensity peak appearing to the left of the LiBH_4_ (narrow) and MgH_2_ (broadened) peaks is present. This peak was selected for generating the edge thresholded images. Figure 5 shows an example of the edge thresholding of LiBH_4_ particles in a sample comprised of 1:3 LiBH_4_:MgH_2_ (uncatalyzed). Figure 6 shows the interfacial volume calculated for each of the single phase powders, *i.e*., LiBH_4_ and MgH_2_, in their catalyzed and uncatalyzed state. No significant trend in interfacial volume is noted. The standard deviation for uncatalyzed and catalyzed LiBH_4_ is 0.04 and the standard deviation for uncatalyzed and catalyzed MgH_2_ is 0.02. The data plotted in Figure 6 is a ratio of the interfacial boundary volume to bulk volume (and is unitless). The standard deviation is +/−0.04 for uncatalyzed and catalyzed LiBH_4_. The +/−0.04 standard deviation is used for the analysis of trends in the interphase volume ratios for the mixed LiBH_4_ and MgH_2_ systems (shown as error bars in Figure 7).

**Figure 8:**
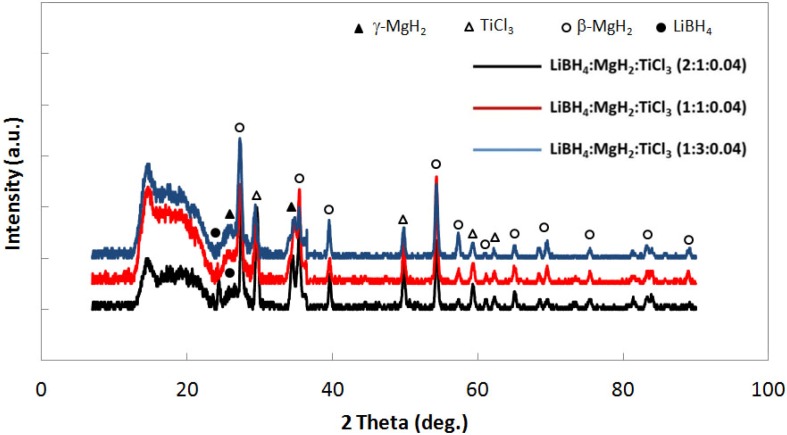
X-ray diffraction for 4 mol % TiCl_3_ catalyzed LiBH_4_:MgH_2_ in 2:1, 1:1 and 1:3 molar ratios.

Figure 7 shows interphase boundary to bulk volume ratios for the mixtures. Both uncatalyzed and catalyzed samples are represented. In all cases, catalyzed systems have higher interfacial volumes relative to the uncatalyzed systems for all molar ratios. These data suggest that the catalyst contributes to reduction in particle size in the mixed powder systems. The reason for this enhanced reduction in particle size is yet unknown, however, it may be attributed to particle embrittlement associated with diffusion Ti^3+^ cations into individual LiBH_4_ or MgH_2_ particles. Still, a different explanation for the increased interfacial boundary to bulk volume ratio within the catalyzed samples might be that TiCl_3_ remained as a separate powder phase in the system. Although comprising only 4 mol. percent of the powder sample, X-ray diffraction data for the catalyzed samples (shown in Figure 8) reveals a peak at 2θ~49.84° which could correspond to either TiCl_3_ or to LiBH_4_ (having a peak at 2θ~49.98°). It is, however, unlikely that the inclusion of TiCl_3_ (as a separate powder phase) is solely responsible for the variation in interphase to bulk volume ratio observed because it was added in only 4 mol % amounts.

The parameter of interfacial boundary volume-to-bulk volume is an important parameter for any solid-solid phase transformation. The interphase boundary volume would indicate the available volume for solid-solid reactions to occur. Here, the pixels found along the edges of the LiBH_4_ particles are highlighted and counted via image analysis. However, this edge count is limited to the light grey LiBH_4_ phase and in no way quantitatively determines the amount of interphase between LiBH_4_ and MgH_2_. For example, the method used for processing this image data does not exclude the possibility that a LiBH_4_ particle is in contact with void space or with another LiBH_4_ particle. Here, it can only be assumed that each LiBH_4_ particle is surrounded by MgH_2_ particles. At low molar fraction of LiBH_4_ in the powders (*i.e*., molar fractions below the percolation threshold where LiBH_4_ particle-to-particle contact occurs)—this is a believed to be a valid assumption.

Other studies have been undertaken to use microtomography for understanding solid-to-solid microstructural and phase transformations. Many studies focus on sintering and neck formation in heterogenous systems [9,10]. Fewer studies have undertaken, as this one does, a comprehensive analysis of interfacial volume driven reactions in heterogenous systems [11].Furthermore, the tomographic imaging allowed (with relative ease) the preparation of samples without exposure to air and moisture known to oxidize or decompose many complex metal hydrides. A detailed image analysis and high spatial resolution of the type presented here would not be possible using a scanning electron microscope unless that microscope is equipped with an environmental sample loading chamber.

## 3. Experimental Section

Magnesium hydride (MgH_2_) and hydrogen storage grade lithium borohydride (LiBH_4_) were obtained commercially from Sigma Aldrich^®^ (St. Louis, MO). Mixtures of the powders were prepared in 1:3, 1:1, 2:1 LiBH_4_:MgH_2_ molar ratios and high energy ball milled for 5 minutes using a SPEX Certiprep 8000M mixer mill and stainless steel milling media. The milling was done within a stainless steel mill jar using two stainless steel balls of 0.5 cm in diameter as milling media. The milling media-to-hydride powder mass ratio was maintained close to 10:1. To study the effect of 4 mol % TiCl_3_ catalyst, a second set of mixtures in 1:3, 1:1, and 2:1 ratios were prepared with catalyst added and were milled for 5 minutes. To prepare control samples, single phase LiBH_4_ and single phase MgH_2_ were both milled for 5 minutes using catalyzed (4 mol % TiCl_3_) and uncatalyzed conditions. Samples were loaded to tapping density into 1mm and 1.87 mm inner diameter polyamide tubes (Goodfellow Corp, Huntingdon, England) and edges were sealed using epoxy putty for transport to the x-ray beamline. (Analysis reported here includes only samples loaded into the 1.87 mm inner diameter tubes because very sparse sample amounts were able to be loaded into the 1 mm diameter tubes.)

Samples were measured using the microtomography instrument located at 2BM Advanced Photon Source (Argonne National Laboratory, IL, USA) [7]. The instrument can reach a 200 nm spatial resolution but was operated at a 2 μm resolution for these experiments. The instrument at 2BM is made for high throughput measurement and is equipped with an automatic sample changer to image up to 20 samples per hour. Samples comprised of an abundance of low atomic number elements are a challenge for absorption based imaging so the sample to detector distance was offset to enhance phase contrast imaging for the LiBH_4_ phase. Data processing was performed using the Avizo^TM^ Software package available at the Louisiana State University’s Center for Computation and Technology.

## 4. Conclusions

Overcoming kinetic limitations are the key to meeting performance targets for a most promising class of metal hydride materials—the destabilized hydrides. The present study focuses on the MgH_2_-destabilized LiBH_4_ system and the effect of transition metal catalysts on those systems. This work demonstrates that three dimensional imaging is poised to be a platform technology in understanding the influences on H_2_ desorption and uptake rates of (i) domain sizes (*i.e*., reactant and product phase particle sizes), (ii) H_2_ adsorption and desorption reaction site density (*i.e*., content of interphase boundary volume per unit sample volume), and (iii) diffusional transport throughout the composite system. Future studies should include examination of powders after desorption excursions. Tomographic 3D imaging can be used to provide much needed data on the relationship between morphology and the kinetics of H_2_ desorption and uptake in the destabilized hydride systems.
